# A Preliminary Investigation into Ridden Water Submersion Training as an Adjunct to Current Condition Training Protocols in Performance Horses

**DOI:** 10.3390/ani11092629

**Published:** 2021-09-07

**Authors:** Claire O’ Brien, Josephine Pegg

**Affiliations:** 1 Department of Biological Sciences, University of Portsmouth, Portsmouth PO1 2DT, UK; 2 Higher Education Department, University Centre Sparsholt, Winchester SO21 2NF, UK; josie.pegg@sparsholt.ac.uk

**Keywords:** horse, equine training, equine fitness, equine exercise physiology, ridden water submersion training, tendon injury, infrared thermography, condition training

## Abstract

**Simple Summary:**

Superficial Digital Flexor Tendon (SDFT) injuries are the most common musculoskeletal injury reported in equestrian jumping disciplines. In an attempt to reduce incidences of injuries in elite event horses, Ridden Water Submersion Training (RWST) is a form of condition training that involves submerging the horse up to sternum height in water and trotting for set intervals. It is used by a small number of trainers to increase cardiovascular fitness whilst potentially minimising tendon temperature increase, which is typically reported during traditional condition training sessions. The results of this study suggest that RWST acted as a moderate sub-maximal intensity level of exercise in a group of elite international event horses whilst preventing the accompanying increase of distal limb temperature commonly associated with condition training on land. RWST could thus be a useful adjunct to current condition training protocols, particularly for horses that compete in disciplines that have high incidence rates of tendon injury. However, further research is required to provide a more comprehensive understanding of the workload imposed during RWST training.

**Abstract:**

This observational study aimed to elucidate the effects of RWST on the cardiovascular and musculoskeletal systems of horses and concurrently determine whether RWST limits distal limb temperature increases previously reported during gallop training on land. A group of 15 clinically sound international event horses were recruited, and heart rate (HR), speed (km/h) and thermal images of the distal limb were analysed at set intervals during RWST training. Intervals of RWST produced a total mean HR_max_ value of 65.18 ± 3.76%, which is within the parameters for increased aerobic stamina. Mean HR increased significantly (*p* < 0.01) while mean distal limb temperature decreased significantly (*p* < 0.01) between warm-up and RWST, which contrasts with positive correlations previously reported during gallop training on land. These preliminary results suggest that RWST can be classed as a moderate submaximal intensity exercise in elite international event horses whilst restricting an increase in temperature of the distal limb that is commonly associated with tendon rupture. Horses competing at very elite levels of eventing only represent a small percentage of the total performance population; therefore, further research is needed to ascertain the physiological effects of RWST in non-elite performance horses, as well as horses competing across various equestrian disciplines.

## 1. Introduction

Tendon and ligament damage in the distal limb occur commonly in performance horses with superficial digital flexor tendon (SDFT) injuries accounting for the majority of injuries reported in the equestrian disciplines of show jumping, eventing and National Hunt racing [1,2,3,4]. In an attempt to reduce incidences of distal limb injuries during training, various water-based training protocols are growing in popularity. Water treadmills (WT) are used for rehabilitation purposes [5,6] but have also recently been employed for the purposes of conditioning unfit competition horses returning to training [7]. Swimming is also utilised in some training programmes with a view to mimicking workload while minimising strain on the limbs by submerging a horse in a swimming pool and allowing the horse to swim at its ‘preferred’ speed [8]. However, a consensus on protocols when using these forms of water-based training methods is yet to be, or only very recently been, established [9,10].

Ridden Water Submersion Training (RWST) is a ridden form of condition training that involves submerging the horse up to sternum height in the water and trotting for set intervals at a pace controlled by the rider. Training can be carried out at the beach or an artificial lake. RWST is selectively used to improve the fitness of elite performance horses that compete up to the Olympic level in the discipline of eventing (Donckers, personal communication, 22 July 2012) and racing [11]. There is currently no scientific evidence to elucidate the physiological effects of this particular form of training, and similarly to the aforementioned water-based training protocols, routines can vary according to the facilities available and the personal preference of trainers. The anecdotal basis behind RWST is derived from the use of topically applied cold treatment (cryotherapy) to prevent/treat musculoskeletal injuries [12,13,14,15] post-exercise by inhibiting inflammatory processes associated with tendon pathologies [16], which occur most commonly (97–99% of cases) in the forelimb [17,18]. Studies have established a linear relationship between increasing tendon temperature and cell damage in both horses [19,20,21] and humans [22], presumably due to repetitive mechanical stresses placed upon the limb during intense activities such as galloping and jumping [4,23]. Repeated or prolonged exposure to these hyperthermic conditions subsequently leads to a higher risk of injury [4,24]. 

Given that the tendon is a poorly regenerative tissue, prompt detection at the early stages of pathology and timely adaptations to training protocols can have a greater outcome than advances in treatment. As such, interest is recently emerging for the application of infrared thermography (IRT) as an early screening tool due to its non-invasive nature and ability to promptly assess skin temperature, which is particularly beneficial when monitoring athletes in training [25,26]. The use of IRT to assess skin temperature is increasing in clinical settings [27] as it has consistently been demonstrated as an early screening tool for inflammatory injuries in humans [28,29,30] and in the evaluation of cryotherapy protocols [31,32,33,34]. While IRT cannot reveal specific pathologies, it facilitates the localisation of increased heat (inflammation) [35], commonly referred to as ‘hot spots’. In horses, IRT has been used to detect early signs of injury before clinical symptoms manifest [36] and to monitor musculoskeletal adaptations to training [26]. With the increasing use of IRT in horses, several studies have reviewed its efficacy as an early screening tool [16,37,38,39,40]. 

The lack of insulating tissues overlying the tendons in the distal limb also has the potential to provide a particularly accurate representation of internal temperature. Despite the high frequency of injuries, however, the distal limb has received limited IRT research. In contrast to human studies [27], equine studies still lack clarification on the ‘standard body position’ of the distal limb. Inter-analyser results are less consistent in the distal limb compared to the whole-body view [38]. The appropriate method of selecting a region of interest (ROI) may thus require further clarification. Equine studies commonly use spot and/or line measurement tools included in basic analysis software. However, spot measurements may not be applicable to the various locations that tendon injuries can occur in the distal limb [41]. Line measurements restrict the ability of IRT to determine thermal ‘hot spots’, as well as compromising reproducibility when mapping line measurements to the estimated locations of underlying structures. Therefore, offline analysis of thermal images using a box measurement of the ROI to define a standardised rectangle covering the soft flexor tissue structure [42] has been employed in this study; the authors believe this method may prove particularly relevant when evaluating general temperature changes in the distal limb. 

The aim of this study was to investigate the effects of RWST to determine whether this form of training would be a suitable adjunct to current training protocols, particularly for horses who participate in disciplines that involve a high risk of tendon injury. We intend to determine the approximate workload of RWST by measuring the cardiovascular response in performance horses during training. Secondly, we aim to monitor skin temperature responses of the distal limb throughout the different stages of training using IRT as a pseudo temperature for distal limb temperature.

## 2. Materials and Methods

Ethical approval was obtained from the Science Faculty Ethics Committee at the University of Portsmouth prior to data collection in April 2018.

### 2.1. Animals

Fifteen clinically healthy registered sport horses (ten geldings and five mares; 12 BWP, 1 ZANG, 1 KWPN and 1 ZVCH) trained for international eventing were recruited from “Donckers Stables” (Antwerp, Belgium). They were housed in individual stalls with common management conditions at the training facility, and previous injuries and competition history were recorded. Horses varied in age (range: 7–14 years old; median: 10 years old) and international competition experience (3*-5* international eventing level). Horses were subject to training procedures in preparation for international competition at the time of data collection. Condition training was completed routinely every sixth day of the training programme, with RWST selected by the trainer as the main form of condition training on these days. Interval training on land was also intermittently used as an alternative form of training. Horses underwent daily soundness evaluations and weekly veterinary screening checks, and any who suffered a tendon injury within the previous six months were excluded [3], as were those who wore stable bandages or had abnormal thermal patterns or asymmetries (>1.25 °C between limbs) [26]. All horses were familiarised with the research equipment prior to the initiation of the study such that no behavioural changes were observed upon use.

### 2.2. Training

Data collection took place at a private training venue in Antwerp, Belgium, on five separate days between April 2018–April 2019. Horses were tacked up (saddle, breastplate and cross country boots) and transported by horsebox to the training venue for the RWST training session (approximately ten-minute journey time). Horses were ridden by their usual riders (n = 5) as assigned by the main rider. All horses undertook the same warm-up of progressively walk, trot, and canter across both flat and inclining/declining terrain (24.51 ± 4 min). The absolute speed of paces varied slightly between individual horses; however, riders worked to ensure the paces were consistent and active for each horse. Depending on the current fitness level and competition level that horses were being prepared for during the time of data collection (3*-5* international eventing level), horses were assigned to either 3 × 3 min (n = 11) or 3 × 4 min (n = 4) RWST intervals by the main rider post-warm-up. Horses were submerged to the depth of the ventral body at the base of the sternum and trotted with 1-min walk breaks on land between each interval. Cross country boots (Kentucky Eventing boots) were removed for cool down in the water in free walk (9.5 ± 3.27 min).

### 2.3. Data Collection

**Figure 1:**
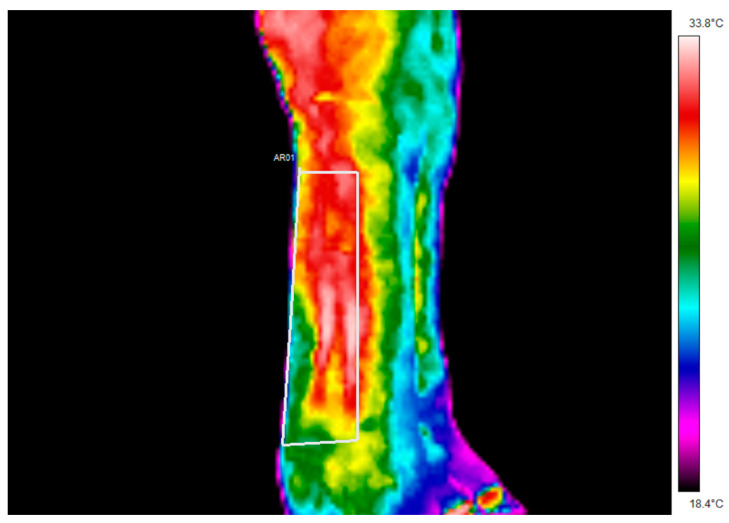
An example of the image of the right lateral forelimb of a subject using infrared thermography. The white frame (AR01) used to create regions of interest includes the palmar aspect of the distal limb, from the top of the third metacarpal bone to just above the proximal sesamoid bone.

Skin temperature of both the left and right forelimbs was measured using an FLIR E8 infrared thermal (IRT) imaging camera (FLIR Commercial Systems Inc., Nashua, NH, USA) in accordance with published guidelines [27,39] as a pseudo-temperature for tendon temperature. The camera was calibrated within 12 months of use. Data were analysed via dedicated software (ThermaCAM™ Researcher, ThermaCAM™ Researcher, FLIR Systems, West Malling, UK) to allow offline (box measurement) analysis of the region of interest (ROI). Wet Bulb Globe Temperature (WGBT) was measured using a Heat Stress WGBT Meter (Extech Model HT30, ExTech Instruments, FLIR Commercial Systems Inc., Nashua, NH, USA). The IRT camera was turned on for a minimum of 20 min in each condition to allow sensor stabilisation following the manufacturer’s guidelines. Emissivity settings of 0.98 were reported [39]. Markers were used to ensure that the distance between the limb and camera was consistent. Three thermal images were taken of each forelimb (Figure 1) [39] whilst the horse was restrained by a handler, ensuring the limbs had equal weight distribution [43]. 

Basal measurements (AM) were recorded in each horse’s stable between 06.00–07.00 before the yard routine commenced. This ensured that factors such as exercise, exposure to direct sunlight, and acclimatisation to the ambient temperature were standardised amongst all horses. Skin temperature of the distal limb was measured again upon arrival at the training venue (Arr), immediately after warm-up (post-WU), immediately after RWST (post-RWST), and immediately after cool-down (post-CD). Water temperature was measured using a HANNA waterproof multiparameter meter (HI9828, HANNA Instruments Ltd., Bedfordshire, UK). The lorry was parked away from direct sunlight and prevailing winds at all times, and thermal images were taken in the middle partition of the stalled horse compartment against black rubber partition dividers. To obtain post-RWST and post-CD readings, limbs were towel-dried immediately after completion, dabbing excess water away while ensuring not to rub the leg and cause friction [27]. 

Polar V800 Equine Heart rate monitors (Polar Electro Oy, Kempele, Finland) were used to measure HR (beats min^−1^), speed (km/h), pace, and altitude (m). Horses were fitted with HR electrode bases as per the manufacturer’s guidelines. Riders wore the watches and commenced the training session once mounted. HR data were downloaded onto the Polar Flow software programme (Polar Electro Oy, Kempele, Finland) and raw data exported onto Microsoft Excel.

### 2.4. Statistical Analysis

**Figure 2:**
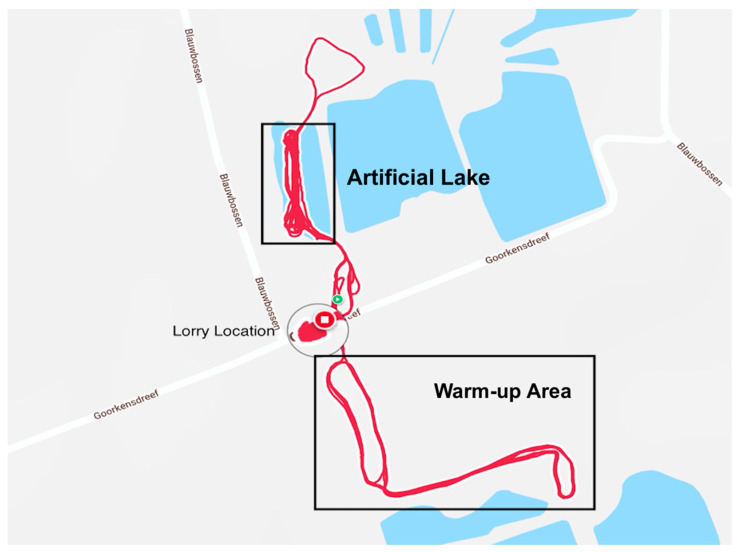
Map data; training venue highlighting the location of the lorry and warm-up area (WU), and the locations for RWST and CD (artificial lake).

Statistical analysis was carried out using IBM SPSS (version 25, SPSS Inc., Chicago, IL, USA). The significance level was set at *p* < 0.05. Data were analysed for normal distribution (Shapiro-Wilk’s test). Mean speed and HR values were calculated for WU, RWST (interval breaks not included), and CD. Start and finish times within each condition were estimated based on the GPS location where horses entered into each area (Figure 2).

HR_max_ is the age-related number of beats per minute of the heart when it is working at maximum capacity and is used to minimise the effect of inter-group variance. An estimation of HR_max_ (adapted for age) can be attained using the formula:

Individual horse’s HR_max_ (bpm) = 240 (bpm) − horses’ age (years)

Mean HR was subsequently converted to mean %HR_max_ using the formula [44]:

%HR_max_ = mean HR (bpm) ÷ individual horse’s HR_max_ (bpm) × 100,

Three-way mixed ANOVA’s were used to test for statistically significant differences in mean HR/HR_max_ responses between the WU, RWST, and CD phases of the training sessions within the group (n = 15). Post hoc analyses (Bonferroni adjustment) were conducted to determine whether between-subject factors (rider, competition experience, age, intensity level of RWST, day of training) had a significant effect on the results. 

Minimum, maximum, and mean temperatures were manually recorded from the rectangular ROI selected [42] in each image (as shown in Figure 1). Left and right forelimb mean temperatures were then combined to give a mean value for every horse under each condition. Due to technical errors with the equipment used to specifically measure WGBT on day one (n = 3), IRT data collected in the AM, Arr, and post-WU conditions were not included in the data analyses that specifically investigated any relationship between distal limb temperature and WGBT.

A Pearson’s Correlation was conducted to determine the strength and direction of a linear relationship between WGBT/H_2_O Temperature and distal limb temperature within each of the five conditions. Three-way mixed ANOVA with post hoc analysis (Bonferroni adjustment) was conducted to determine whether there was a statistically significant difference in mean distal limb temperature between all conditions within the group and to test the effect of between-subject factors (rider, competition experience, age, intensity level of RWST, day of training) on the results. Multiple regression analysis was subsequently conducted on the post-work (PWK) condition only to explore the relationship between distal limb temperature response according to water/WGBT temperature, HR, day, horse’s age and competition experience. Multiple regression analysis was also conducted to predict distal limb temperature response in the two water-based conditions, PWK and post-cool down (PCD), according to condition, water/WGBT temperature, HR, boots, and speed.

## 3. Results

### 3.1. Training Sessions

Data were collected from five training sessions; in each session, three different horses were observed once each (n = 15). The sessions comprised of a standardised warm-up (7.53 ± 0.62 km/h) followed by the main RWST session that consisted of three intervals of trot (8.15 ± 0.57 km/h) with 1 min rest breaks walking on land and a cool-down period of walking in the water (3.87 ± 0.43 km/h). Data was normally distributed (*p* > 0.05). Condition did not show statistically significant changes in speed between WU and RWST, but elicited significant changes in CD, F(2, 14) = 237.279, *p* < 0.001, partial η2 = 0.987, with speed increasing from WU (7.53 ± 0.62 km/h) to RWST (8.15 ± 0.57 km/h) and significantly decreasing CD (3.87 ± 0.43 km/h).

### 3.2. Cardiovascular Response to Training Sessions

**Figure 3:**
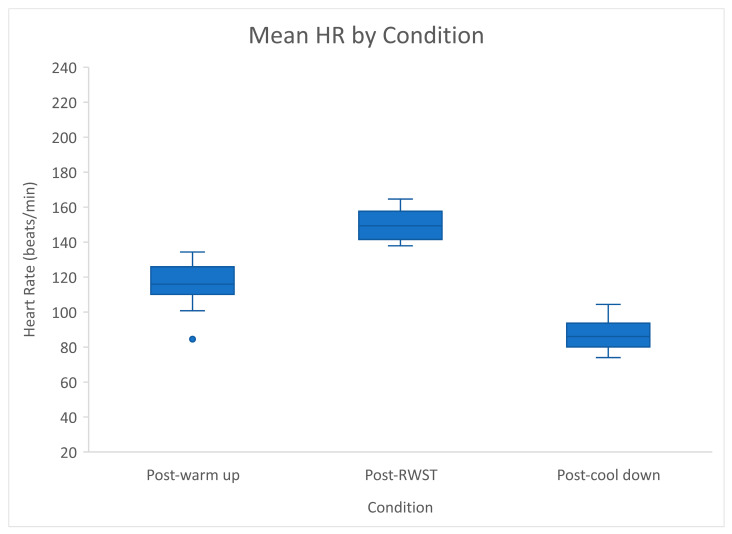
Mean HR by active condition—error bars represent standard deviation and isolated point represents outlier.

HR was normally distributed across the three active conditions (WU, RWST, and CD; *p* > 0.05). Each condition elicited statistically significant changes in mean HR, F(2, 26) = 280.983, *p* < 0.001, partial η2 = 0.96. There was a statistically significant mean increase in HR of 34.8 beats/min (95% CI [26.49, 42.237], *p* < 0.01) from WU (114.97 ± 12.12 beats/min) to RWST (149.77 ± 8.26 beats/min), and a statistically significant mean decrease of 63.16 beats/min (95% CI [55.16, 69.76]) in CD (86.61 BPM ± 9.13 beats/min). HR values of all horses specifically during RWST ranged from 137 bpm–165 bpm (refer to Figure 3). There was a strong statistically significant positive correlation between HR and speed in the conditions RWST and CD, r (28) = 0.935, *p* < 0.001, with speed explaining 87% of variations in HR.

**Figure 4:**
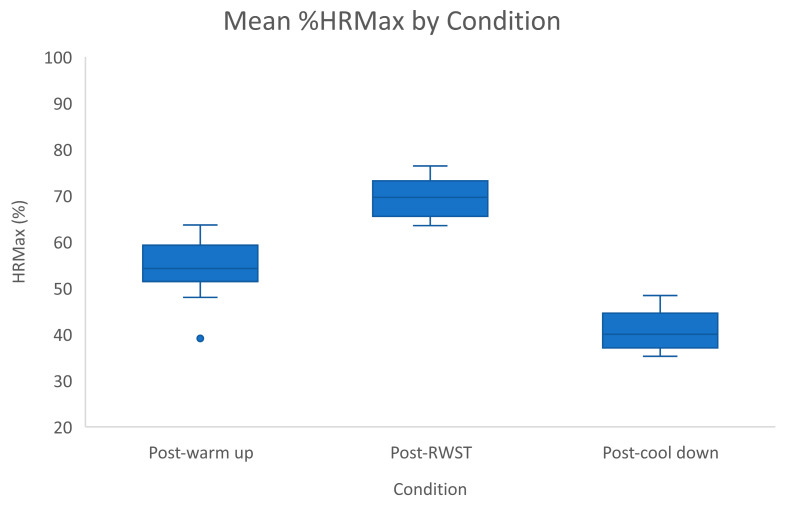
Mean %HR_max_ by active condition—error bars represent standard deviation and isolated point represents outlier.

HR was converted to %HR_max_ for each horse (see Figure 4). Mean %HR_max_ was also statistically significant across the three active conditions, F(2, 28) = 305.931, *p* < 0.001, partial η2 = 0.956. There was a statistically significant increase of 15.14% (95% CI [12.86, 19.68], *p* < 0.01) in mean %HR_max_ from WU (50.04 ± 5.31%) to RWST (65.18 ± 3.76%), and a statistically significant mean decrease of 27.07% (95% CI [−32.3, −25.88]) to CD (38.11 ± 3.89%).

In terms of between-subject factors, there was a statistically significant three-way interaction between rider, competition experience and condition on %HR_max_, F(2,14) = 9.499, *p* = 0.01, partial η2 = 0.576. Statistical significance of a simple main effect was accepted at a Bonferroni-adjusted alpha level of 0.025. There was a statistically significant simple main effect of rider in the WU condition only, F(3, 7) = 10.9, *p* = 0.005, but not in RWST, F(3, 7) = 3.304, *p* = 0.087, or CD, F(3, 7) = 0.667, *p* = 0.599.

### 3.3. Distal Limb Temperature Response to RWST

**Figure 5:**
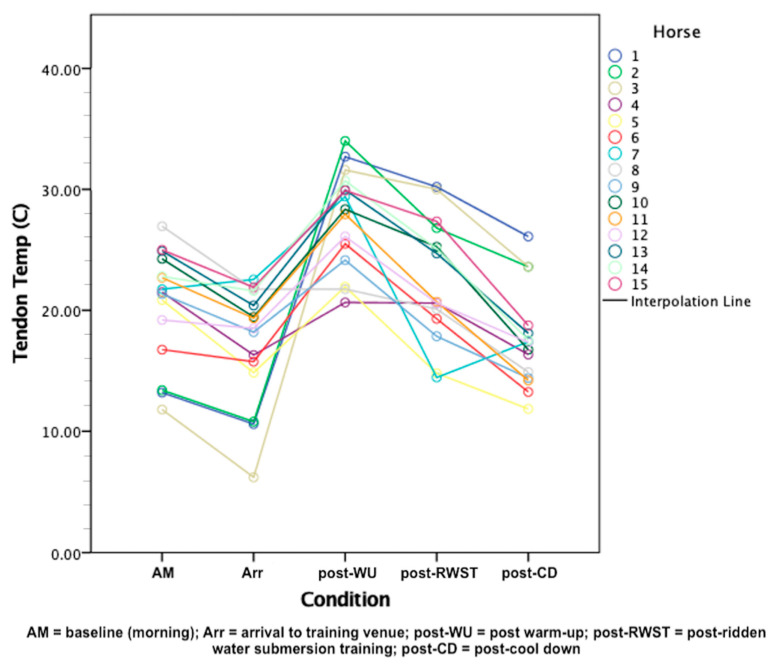
Distal limb temperature recorded per horse by condition.

Distal limb temperature data was normally distributed across the five conditions (*p* > 0.05). Mean distal limb temperatures across the five conditions can be viewed in Figure 5 below.

There was a strong significant positive correlation between WGBT and distal limb temperature baseline measures in the morning (4.47 ± 1.29 °C; r = 0.63, *p* = 0.027), with WGBT explaining 40% of the variation in distal limb temperature in this condition. There was no statistically significant correlation between WGBT and distal limb temperature post-arrival to the training venue (6.11 ± 2.69 °C; r = 0.47, *p* = 0.127), with WGBT explaining 22% of the variation in distal limb temperature in this condition. There was no statistically significant correlation between WGBT and distal limb temperature post-WU (6.11 ± 2.69 °C; r = 0.15, *p* = 0.648), with WGBT explaining 2% of the variation in distal limb temperature. For conditions RWST and CD (N = 15), there was a strong significant positive correlation between H_2_O temperature and RWST (7.69 ± 5.42 °C; r = 0.71, *p* = 0.03), with WGBT explaining 50% of the variation in distal limb temperature, and a strong significant positive correlation between H_2_O Temp and CD (7.69 ± 5.42 °C; r = 0.69, *p* = 0.04), with WGBT explaining 48% of the variation in distal limb temperature.

Conditions WU, RWST, and CD elicited statistically significant changes in distal limb temperature F(4, 20) = 37.715, *p* < 0.001, partial η2 = 0.883, with distal limb temperature decreasing from AM (20.43 ± 4.66 °C) to Arr (17.21 ± 4.84 °C), increasing post-WU (27.64 ± 4.14 °C), and decreasing post-RWST (22.52 ± 4.99 °C) and post-CD (17.61 ± 4.04 °C). Post hoc analysis with a Bonferroni adjustment revealed that distal limb temperature statistically significantly decreased from AM to Arr (3.055 °C, 95% CI [0.23, 6.09 °C], *p* < 0.001), and statistically significantly increased from AM to post-WU (9.299 °C, 95% CI [1.86, 16.74 °C], *p* < 0.019). Distal limb temperature was not statistically significant from AM to post-RWST (3.723 °C, 95% CI [−2.22, 9.67 °C], *p* = 0.304) or post-CD (−1.016 °C, 95% CI [−6.25, 4.22 °C], *p* = 1.000).

A multiple regression analysis was subsequently carried out to predict distal limb temperature response from condition, water/WGBT temperature, HR, day of training, horse age, competition experience and rider in the conditions WU, RWST, and CD. Condition and water/WGBT temperature were found to statistically significantly predict distal limb temperature response during the three conditions, F(7,37) = 16.285, *p* = 0.01. R^2^ for the overall model was 75.5% with an adjusted R^2^ of 70.9%, indicative of a large size effect according to Cohen [45]. The same analysis was carried out to predict distal limb temperature response from H_2_O temperature, HR, boots, and speed in the two water-based conditions, RWST and post-CD (n = 15). All five variables added statistical significance to the prediction, F(4,25) = 12.872, *p* = 0.01. R^2^ for the overall model was 67.3%, with an adjusted R^2^ of 62.1%.

## 4. Discussion

To the authors’ knowledge, observations made in this preliminary study confirm for the first time that RWST can be classed as a moderate submaximal intensity exercise in elite event horses [46]. The use of HR monitors can offer a reliable indicator of cardiovascular workload and fitness in horses [47]. Data recorded during RWST intervals produced a total mean HR_max_ value of 65.18 ± 3.76%, which, while sub-maximal, is within the estimated parameters for maintaining the current level of aerobic stamina in horses used in this study [46], with 75–85% HR_max_ reaching anaerobic threshold [47]. HR/HR_max_ values decrease with increasing fitness, and the horses used in this study were already at peak fitness for elite international competition. As such, further research needs to be carried out to ascertain the effects of RWST on a more representative sample of the total equine performance population, i.e., in (1) non-elite performance horses and (2) across a range of disciplines. Furthermore, whether RWST resulted in subsequent changes in fitness level cannot be objectively assessed with this study design. This would require evaluations under maximal conditions, which was not a viable option with this sample of horses. Instead, proxy values were calculated to determine approximate HR_max_ values, which have limited application beyond the preliminary findings. Therefore, any comments regarding the potential effects of RWST on fitness in this paper are speculation by the authors. Environmental factors which also may have influenced HR results include water temperature and the effects of water density on respiration, which are discussed further below.

Age, fitness, and competition level determined the chosen intensity of the training sessions by the main competition rider (either 3 × 3 min intervals or 3 × 4 min intervals). HR_max_ values between the two groups did not differ significantly, suggesting that horses were trained at a level appropriate for their current fitness level and experience. Similar to training on land [8], HR also correlated positively with speed in the water; however, the magnitude of the response is less pronounced in water due to restrictions on speed—as previously shown in horse and human studies [8,48]. The increased resistance when training in water was evident—the mean speed between WU on land (7.60 ± 0.60 km/h) and RWST (8.27 ± 0.69 km/h) was within 1 km/h, and yet the cardiovascular response to RWST was significantly higher. This is likely due to the increased density of the water, which affects both the level of resistance against the musculoskeletal system during locomotion (i.e., limiting limb protraction and altering stride pattern [10]) as well as the respiratory system. This is further supported by WT studies which showed that a high degree of resistance created by water increased workload whilst reducing ground reaction forces in both horses [6,7,9,49,50] and dogs [49]. With regard to the effects of water density on respiration, patterns during swimming have previously been shown to increase expiratory time and pressure significantly [50], turning exhalation from a passive to an active process. In contrast, the ratio of expiratory/inspiratory duration was not significantly influenced in a water treadmill up to sternum height in walk mode when compared to control values (no water) [9]. The researchers cannot currently speculate on how RWST influences the breathing strategy of horses during training, most likely due to variations in workload and depth of submersion when compared to previous studies. Whilst RWST may subsequently be a safer form of training for the horse’s musculoskeletal system, due to the hydrostatic buoyancy in water, further research is warranted to ascertain whether the breathing strategy of the horse differs significantly from other forms of training and how this may affect respiration rates during training. 

It must also be noted that water density varies with temperature [48], which is reported to have a direct linear relationship with speed, HR response, and plasma lactate concentration during swim performance [8,51,52,53]. Fluctuations in maximal metabolic power (as suggested by the decrease or increase in plasma lactate) are possibly due to changes in muscle temperature as a result of varying temperatures, which subsequently affect biochemical and functional processes of the working muscles [51]. Varying water temperatures across different training days did not have a significant effect on HR or speed in this study, but this was likely due to the small sample size used. Therefore, it may be beneficial to investigate the effect of varying water temperatures on speed and HR response in the future. Practically speaking, the authors also recommend that trainers should monitor the effects of training objectively using HR monitors as opposed to aiming for an approximate speed due to fluctuations in water temperature [54]. 

With regard to the effects of RWST on distal limb temperature, mean distal limb temperature fluctuated significantly throughout the five conditions. WGBT/H_2_O temperature correlated positively in all conditions, with statistically significant correlations in the AM, post-RWST, and post-CD conditions. For the AM condition, our findings support previous studies, which also found a positive correlation at rest between ambient temperature and absolute joint temperature [55]. In conditions Arr and post-WU, the non-significant results indicate the overriding effect of other extraneous factors in these conditions, such as transit and increased physical activity during warm-up.

The positive correlations between H_2_O temperature and the RWST and CD conditions support the concept that H_2_O temperature had an overriding effect on distal limb temperature, irrespective of other extraneous factors in these conditions. Therefore, in contrast to HR response, distal limb temperature continually decreased regardless of the increased cardiovascular demands of RWST between the three conditions. Previous studies have found a mean increase in tendon temperature of 2.5 °C min^−1^ during gallop with mean peak temperatures attained in the tendon central core of 43.3 °C [21] and 45 °C [19]. Such values are thought to occur due to the excess production of heat as the tendon extends and contracts repeatedly [21,56,57]. This form of training may subsequently assist in reducing distal limb temperature increases that commonly precede tendon rupture during more traditional forms of condition training on land.

The authors cannot confirm whether the decrease in distal limb temperature during RWST is due to the water temperature, as it was similar to the WGBT temperature on some days. Submerging horses in water up to the ulna results in a 10.5% reduction in bodyweight and, up to the point of shoulder height, reduces bodyweight by up to 31.3% [58], which is also likely to significantly reduce loading on the distal limb during RWST. Submerging horses in water has also been shown to significantly alter range of motion (ROM) of joints [56,59], increase stride length [57], decrease stride frequency [57,60], and increase the duration of the swing phase [59]. While it is still not clear which factors directly influence the reduced biomechanical strain of RWST, it is evident that this form of training does not elicit the same strain response as equivalent condition training methods on land. RWST may reduce degenerative changes observed preceding tendinosis, where repeated strain values above particular intensities lead to accumulation of microdamage [16,17], ultimately leading to tendon rupture.

## 5. Conclusions

Preliminary results from this observational study have established for the first time that RWST can be classed as a moderate submaximal intensity exercise in elite event horses whilst restricting an increase in temperature of the distal limb that is commonly associated with tendon rupture. Due to the study design utilised and the sample of horses selected, the researchers were limited to using non-invasive measures available to measure the physiological effects of RWST and could not carry out evaluations of fitness level under maximal conditions before commencing data collection. As such, the authors utilised proxy values for HRmax in order to estimate fitness levels in horses, and therefore, cannot comment objectively on whether this level of work resulted in changes to fitness levels. Therefore, any comments regarding the potential effects of RWST on fitness in this paper are speculation by the authors. As such, further research is required to properly establish the long-term effects of incorporating RWST into a training programme, as well as various factors that can affect physiological responses. Since the data collection was completed, recent advances in portable respirometry systems have been validated [9,61] and are also likely to play a particularly important role in determining workload more comprehensively under such field conditions in the future. 

Preliminary data show that RWST may be a useful adjunct to current condition training methods when training horses for competition, especially with regard to disciplines that involve a high risk of distal limb injury. In contrast to the increase in cardiovascular response observed between WU and RWST, distal limb temperature was negatively correlated with HR response between these conditions. This suggests that RWST may prevent an increase of tendon temperature commonly associated with increased cardiovascular demands during training on land. Combining the use of IRT with other modalities such as ultrasound tissue characterisation (UTC) may also increase the reliability of such measurements in future studies [23,62]. 

The mechanisms that prevent an increase in distal limb temperature during RWST require further elucidation in relation to the reduced loading placed upon the distal limb. WT studies may be able to further clarify how different combinations of water height and temperature affect distal limb temperatures post-exercise. As with previous research in WT, further investigations on the long-term effects of RWST on ROM, stride length, and stride frequency would also be beneficial due to alterations in stride patterns observed during training.

In conclusion, further investigation into RWST is warranted to establish a more robust understanding of the effect of this type of condition training currently used in both elite and non-elite performance horses and potentially decide at what stage of the fitness programme RWST could be incorporated. 

## Data Availability

Datasets used and/or analysed during the current study are available from the corresponding author on reasonable request.

## References

[1] Thorpe C.T., Spiesz E.M., Chaudhry S., Screen H.R.C., Clegg P.D. (2015). Science in brief: Recent advances into understanding tendon function and injury risk. Equine Vet. J..

[2] Egenvall A., Tranquille C.A., Lönnell A.C., Bitschnau C., Oomen A., Hernlund E., Montavon S., Franko M.A., Murray R.C., Weishaupt M.A. (2013). Days-lost to training and competition in relation to workload in 263 elite show-jumping horses in four European countries. Prev. Vet. Med..

[3] Singer E.R., Barnes J., Saxby F., Murray J.K. (2008). Injuries in the event horse: Training versus competition. Vet. J..

[4] Murray R.C., Dyson S.J., Tranquille C., Adams V. (2006). Association of type of sport and performance level with anatomical site of orthopaedic injury diagnosis. Equine Vet. J..

[5] Nankervis K.J., Launder E.J., Murray R.C. (2017). The Use of Treadmills Within the Rehabilitation of Horses. J. Equine Vet. Sci..

[6] Muñoz A., Saitua A., Becero M., Riber C., Satué K., Sánchez De Medina A., Argüelles D., Castejón-Riber C. (2019). The use of the water treadmill for the rehabilitation of musculoskeletal injuries in the sport horse. J. Vet. Res..

[7] Greco-Otto P., Bond S., Sides R., Bayly W., Leguillette R. (2020). Conditioning equine athletes on water treadmills significantly improves peak oxygen consumption. Vet. Rec..

[8] Klomp M., Munsters C.C.B.M., Van Oldwitenborgh-Oosterbaan M.M. (2014). Swimming exercise and race performance in thoroughbred racehorses. Pferdeheilkunde.

[9] Greco-Otto P., Bond S., Sides R., Kwong G.P.S., Bayly W., Léguillette R. (2017). Workload of horses on a water treadmill: Effect of speed and water height on oxygen consumption and cardiorespiratory parameters. BMC Vet. Res..

[10] Nankervis K., Tranquille C., Mccrae P., York J., Lashley M., Baumann M., King M., Sykes E., Lambourn J., Miskimmin K. (2021). Consensus for the General Use of Equine Water Treadmills for Healthy Horses. Animals.

[11] Steel C., Morrice-West A. (2019). A survey of trainers on the use of swimming and other water-based exercise for Thoroughbred racehorses in Australia. Comp. Exerc. Physiol..

[12] Van Eps A.W. (2010). Therapeutic hypothermia (cryotherapy) to prevent and treat acute laminitis. Vet. Clin. North Am. Equine Pr..

[13] Van Eps A.W., Leise B.S., Watts M., Pollitt C.C., Belknap J.K. (2012). Digital hypothermia inhibits early lamellar inflammatory signalling in the oligofructose laminitis model. Equine Vet. J..

[14] Turner T.A. (1991). Thermography as an aid to the clinical lameness evaluation. Vet. Clin. North Am. Equine Pr..

[15] Soroko M., Dudek K., Howell K., Jodkowska E., Henklewski R. (2014). Thermographic Evaluation of Racehorse Performance. J. Equine Vet. Sci..

[16] O’Brien C., Marr N., Thorpe C. (2020). Microdamage in the equine superficial digital flexor tendon. Equine Vet. J..

[17] Kasashima Y., Takahashi T., Smith R.K.W., Goodship A.E., Kuwano A., Ueno T., Hirano S. (2010). Prevalence of superficial digital flexor tendonitis and suspensory desmitis in Japanese Thoroughbred flat racehorses in 1999. Equine Vet. J..

[18] Lam K.H., Parkin T.D.H., Riggs C.M., Morgan K.L. (2007). Descriptive analysis of retirement of Thoroughbred racehorses due to tendon injuries at the Hong Kong Jockey Club (1992–2004). Equine Vet. J..

[19] Yamasaki H., Goto M., Yoshihara T., Sekiguchi M., Konno K., Momoi Y., Iwasaki T. (2001). Exercise-Induced Superficial Digital Flexor Tendon Hyperthermia and the Effect of Cooling Sheets on Thoroughbreds. J. Eq. Sci.

[20] Birch H.L., Wilson A.M., Goodship A.E. (1997). The effect of exercise-induced localised hyperthermia on tendon cell survival. J. Exp. Biol..

[21] Wilson A.M., Goodship A.E. (1994). Exercise-induced Hyperthermia as a Possible Mechanism for Tendon Degeneration. J. Biomechmics.

[22] Cook J.L., Khan K.M. (2007). Etiology of Tendinopathy. Tendinopathy in Athletes.

[23] Docking S.I., Daffy J., van Schie H.T.M., Cook J.L. (2012). Tendon structure changes after maximal exercise in the Thoroughbred horse: Use of ultrasound tissue characterisation to detect in vivo tendon response. Vet. J..

[24] Munsters C.C., van den Broek J., Welling E., van Weeren R., van Oldruitenborgh-Oosterbaan M.M. (2013). A prospective study on a cohort of horses and ponies selected for participation in the European Eventing Championship: Reasons for withdrawal and predictive value of fitness tests. BMC Vet. Res..

[25] Redaelli V., Luzi F., Mazzola S., Bariffi G.D., Zappaterra M., Nanni Costa L., Padalino B., Costa L.N., Padalino B., Nanni Costa L. (2019). The use of infrared thermography (IRT) as stress indicator in horses trained for endurance: A pilot study. Animals.

[26] Prochno H.C., Barussi F.M., Bastos F.Z., Weber S.H., Bechara G.H., Rehan I.F., Michelotto P.V. (2020). Infrared Thermography Applied to Monitoring Musculoskeletal Adaptation to Training in Thoroughbred Race Horses. J. Equine Vet. Sci..

[27] Moreira D.G., Costello J.T., Brito C.J., Adamczyk J.G., Ammer K., Bach A.J.E., Costa C.M.A., Eglin C., Fernandes A.A., Fernández-Cuevas I. (2017). Thermographic imaging in sports and exercise medicine: A Delphi study and consensus statement on the measurement of human skin temperature. J. Therm. Biol..

[28] Dibai-Filho A.V., Guirro E.C.O., Ferreira V.T.K., Brandino H.E., Vaz M.M.O.L.L., de Jesus Guirro R.R. (2015). Reliability of different methodologies of infrared image analysis of myofascial trigger points in the upper trapezius muscle. Brazilian J. Phys. Ther..

[29] Alfieri F.M., Battistella L.R. (2018). Body temperature of healthy men evaluated by thermography: A study of reproducibility. Technol. Heal. Care.

[30] Gómez-Carmona P., Fernández-Cuevas I., Sillero-Quintana M., Arnaiz-Lastras J., Navandar A. (2020). Infrared Thermography Protocol on Reducing the Incidence of Soccer Injuries. J. Sport Rehabil..

[31] Costello J.T., Culligan K., Selfe J., Donnelly A.E. (2012). Muscle, skin and core temperature after −110 °C cold air and 8 °C water treatment. PLoS ONE.

[32] Adamczyk J.G., Krasowska I., Boguszewski D., Reaburn P. (2016). The use of thermal imaging to assess the effectiveness of ice massage and cold-water immersion as methods for supporting post-exercise recovery. J. Therm. Biol..

[33] Silva Y.A., Santos B.H., Andrade P.R., Santos H.H., Moreira D.G., Sillero-Quintana M., Ferreira J.J.A. (2017). Skin temperature changes after exercise and cold water immersion. Sport Sci. Health.

[34] Selfe J., Alexander J., Costello J.T., May K., Garratt N., Atkins S., Dillon S., Hurst H., Davison M., Przybyla D. (2014). The Effect of Three Different (−135°C) Whole Body Cryotherapy Exposure Durations on Elite Rugby League Players. PLoS ONE.

[35] Eddy A.L., Van Hoogmoed L.M., Snyder J.R. (2001). The Role of Thermography in the Management of Equine Lameness. Vet. J..

[36] Michelotto B.L., Rocha R.M.V.M., Michelotto P.V. (2016). Thermographic Detection of Dorsal Metacarpal/Metatarsal Disease in 2-Year-Old Thoroughbred Racehorses: A Preliminary Study. J. Equine Vet. Sci..

[37] Soroko M., Davies Morel M.C.G., Howell K. (2017). The Application of Infrared Thermography in Equestrian Sport. Application of Infrared Thermography in Sports Science.

[38] Howell K., Dudek K., Soroko M. (2020). Thermal camera performance and image analysis repeatability in equine thermography. Infrared Phys. Technol..

[39] Soroko M., Howell K. (2016). Infrared Thermography: Current Applications in Equine Medicine. J. Equine Vet. Sci..

[40] Redaelli V., Bergero D., Zucca E., Ferrucci F., Costa L.N., Crosta L., Luzi F. (2014). Use of thermography techniques in equines: Principles and applications. J. Equine Vet. Sci..

[41] Alzola R., Easter C., Riggs C.M., Gardner D.S., Freeman S.L. (2018). Ultrasonographic-based predictive factors influencing successful return to racing after superficial digital flexor tendon injuries in flat racehorses: A retrospective cohort study in 469 Thoroughbred racehorses in Hong Kong. Equine Vet. J..

[42] Tumilty S., Adhia D.B., Smoliga J.M., Gisselman A.S. (2019). Thermal profiles over the Achilles tendon in a cohort of non-injured collegiate athletes over the course of a cross country season. Phys. Ther. Sport.

[43] Soroko M., Henklewski R., Filipowski H., Jodkowska E. (2013). The Effectiveness of Thermographic Analysis in Equine Orthopedics. J. Equine Vet. Sci..

[44] Vincent T.L., Newton J.R., Deaton C.M., Franklin S.H., Biddick T., Mckeever K.H., Mcdonough P., Young L.E., Hodgson D.R., Marlin D.J. (2006). Retrospective study of predictive variables for maximal heart rate (HRmax) in horses undergoing strenuous treadmill exercise. Equine Vet. J..

[45] Cohen J. (1962). The statistical power of abnormal-social psychological research: A review. J. Abnorm. Soc. Psychol..

[46] Bitschnau C., Jones J., Haldi J., Laukkanen R., Weishaupt M. (2013). POLAR SPORT ZONES FOR HORSES. White Pap.-Polar Sport Zones Horses.

[47] Allen K.J., Young L.E., Franklin S.H. (2016). Evaluation of heart rate and rhythm during exercise. Equine Vet. Educ..

[48] Mougios V., Deligiannis A. (1993). Effect of water temperature on performance, lactate production and heart rate at swimming of maximal and submaximal intensity. J Sport. Med Phys Fit..

[49] Levine D., Marcellin-Little D.J., Millis D.L., Tragauer V., Osborne J.A. (2010). Effects of partial immersion in water on vertical ground reaction forces and weight distribution in dogs. Am. J. Vet. Res..

[50] Hobo S., Yoshida K., Yoshihara T. (1998). Characteristics of Respiratory Function during Swimming Exercise in Thoroughbreds. J. Vet. Med Sci..

[51] Arabas C., Mayhew J.L., Hudgins P.M., Bond G.H. (1987). Relationships among work rates, heart rates, and blood lactate levels in female swimmers. J. Sports Med. Phys. Fitness.

[52] Keskinen K., Komi P., Rusko H. (1989). A Comparative Study of Blood Lactate Tests in Swimming*. Int. J. Sports Med..

[53] Misumi K., Sakamoto H., Shimizu R. (1994). Changes in blood lactate and heart rate in thoroughbred horses during swimming and running according to their stage of training. Vet. Rec..

[54] Williams J., Kenworthy K., Jones T., Marlin D., Tabor G. (2019). The role of heart rate monitoring to assess workload during maintenance interval training in National Hunt racehorses. J. Vet. Behav..

[55] Soroko M., Howell K., Dudek K. (2017). The effect of ambient temperature on infrared thermographic images of joints in the distal forelimbs of healthy racehorses. J. Therm. Biol..

[56] Mendez-Angulo J.L., Firshman A.M., Groschen D.M., Kieffer P.J., Trumble T.N. (2013). Effect of water depth on amount of flexion and extension of joints of the distal aspects of the limbs in healthy horses walking on an underwater treadmill. Am. J. Vet. Res..

[57] Scott R., Nankervis K., Stringer C., Westcott K., Marlin D. (2010). The effect of water height on stride frequency, stride length and heart rate during water treadmill exercise. Equine Vet. J..

[58] McClintock S.A., Hutchins D.R., Brownlow M.A. (1987). Determination of weight reduction in horses in flotation tanks. Equine Vet. J..

[59] Mccrae P., Bradley M., Rolian C., Léguillette R. (2021). Water height modifies forelimb kinematics of horses during water treadmill exercise Abstract. Comp. Exerc. Physiol..

[60] Greco-Otto P., Baggaley M., Edwards W.B., Léguillette R. (2019). Water treadmill exercise reduces equine limb segmental accelerations and increases shock attenuation. BMC Vet. Res..

[61] Sides R.H., Kirkpatrick R., Renner E., Gough K., Katz L.M., Evans D.L., Bayly W.M. (2018). Validation of masks for determination of $\dot{V}$O2max in horses exercising at high intensity. Equine Vet. J..

[62] Plevin S., McLellan J., van Schie H., Parkin T. (2019). Ultrasound tissue characterisation of the superficial digital flexor tendons in juvenile Thoroughbred racehorses during early race training. Equine Vet. J..

